# Crosstalk between Exercise-Derived Endocannabinoidome and Kynurenines: Potential Target Therapies for Obesity and Depression Symptoms

**DOI:** 10.3390/ph16101421

**Published:** 2023-10-05

**Authors:** Tiffany Wences Chirino, Edgar Rangel López, Alexandra Luna Angulo, Paul Carrillo Mora, Carlos Landa Solis, María Alejandra Samudio Cruz, Alim C. Fuentes Bello, Rogelio Paniagua Pérez, Juan Ríos Martínez, Laura Sánchez Chapul

**Affiliations:** 1 Neuromuscular Diseases Laboratory, Clinical Neurosciences Division, National Institute of Rehabilitation “Luis Guillermo Ibarra Ibarra”, Mexico City 14389, Mexico; tiff.wen@ciencias.unam.mx (T.W.C.); abluna@inr.gob.mx (A.L.A.); alim1.fuentes@comunidad.unam.mx (A.C.F.B.); 2 Cell Reprogramming Laboratory, National Institute of Neurology and Neurosurgery “Manuel Velasco Suárez”, Mexico City 14269, Mexico; raledg@hotmail.com; 3 Clinical Neurosciences Division, National Institute of Rehabilitation “Luis Guillermo Ibarra Ibarra”, Mexico City 14389, Mexico; pcarrillo@inr.gob.mx (P.C.M.); msamudio@inr.gob.mx (M.A.S.C.); 4 Tissue Engineering, Cell Therapy, and Regenerative Medicine Unit, National Institute of Rehabilitation “Luis Guillermo Ibarra Ibarra”, Mexico City 14389, Mexico; clanda@inr.gob.mx; 5 Biochemistry Laboratory, National Institute of Rehabilitation “Luis Guillermo Ibarra Ibarra”, Mexico City 14389, Mexico; rpaniagua@inr.gob.mx; 6 Health Sciences Research Institute, Mexican Navy, Mexico City 04470, Mexico; juan_rios_mtz@yahoo.com.mx

**Keywords:** exercise, endocannabinoids, kynurenines, obesity, depression

## Abstract

The kynurenine pathway (KP) and the endocannabinoid system (ECS) are known to be deregulated in depression and obesity; however, it has been recognized that acute physical exercise has an important modulating role inducing changes in the mobilization of their respective metabolites—endocannabinoids (eCBs) and kynurenines (KYNs)—which overlap at some points, acting as important antidepressant, anti-nociceptive, anti-inflammatory, and antioxidant biomarkers. Therefore, the aim of this review is to analyze and discuss some recently performed studies to investigate the potential interactions between both systems, particularly those related to exercise-derived endocannabinoidome and kynurenine mechanisms, and to elucidate how prescription of physical exercise could represent a new approach for the clinical management of these two conditions.

## 1. Introduction

Depression and obesity are highly prevalent conditions with relevant public health implications. In 2016, the World Health Organization (WHO) reported that approximately 2 billion adults were overweight, and 650 million of them were obese [1]. The WHO also reported that 5% of adults suffer from depression, and the alarming increase in this percentage during recent decades highlights the urgency of implementing more effective strategies against these devastating disorders [2]. Depression and obesity hold a bidirectional relationship with each other; that is, the presence of one increases the risk for developing the other, since they are, in many cases, comorbid conditions [3]. Therefore, it is mandatory to continue investigating the mechanisms responsible for the intricate metabolic and physiological pathways associated with both conditions. Particularly, both the KP and the ECS are known to be deregulated in some pathological conditions, including depression [4,5] and obesity, among others [6,7]. Depression and obesity are importantly influenced by physical exercise—defined as a planned, structured and repetitive activity that improves and maintains physical fitness and health—inducing changes in the mobilization of endocannabinoids (eCBs) and kynurenine metabolites (KYNs) [8,9]. eCB mobilization has been related to the activation of eCB receptor 1 (CB1) in the liver and adipose tissue, and a significant synthesis of eCB in skeletal muscle has been reported to regulate metabolism and restore energy following exercise [10]. On the other hand, the peripheric mobilization of KYNs has been related to suppression of KYN accumulation in the central nervous system (CNS) through an activation of KYN clearance in exercised skeletal muscle through the action of kynurenine aminotransferases enzymes (KATs) [11]. Thus, the effects of exercise training observed in obesity and depression models through modulation of these two pathways suggest an undescribed mechanism of crosstalk mediated by these metabolites, as reported in disease contexts like migraine [12]. Furthermore, the biological and biochemical signals responsible for modifying the rates of synthesis and degradation of both KYNs and eCBs, as well as the effects exerted by these molecules, which sometimes converge or overlap, will be described further. Therefore, the aim of this review is to analyze and discuss some studies performed recently to investigate the potential interactions between both systems, particularly those related to exercise-derived endocannabinoidome and kynurenine mechanisms, and to elucidate how the prescription of physical exercise could represent a new approach for the clinical management of these two conditions.

## 2. Modulation of the KP and the ECS through Physical Exercise

The benefits of regular practice of physical exercise for managing the symptoms of depression and obesity are widely accepted [13,14]. The American College of Sports Medicine recommends 150 min per week of moderate-intensity aerobic physical activity, 75 min per week of high-intensity aerobic exercise, or a combination of moderate and intense aerobic activity spread throughout the week, for weight loss and prevention of weight regain for adults [15]. The National Institute for Health and Care Excellence (NICE) guidelines recommend exercise interventions provided by a trained practitioner that include moderate-intensity aerobic exercise adapted to each individual’s needs [16]. Furthermore, exercise interventions have been shown to reduce depressive symptoms in adults and offer an additional treatment or adjuvant therapy [17]. Therefore, a sedentary lifestyle and physical inactivity are associated with high mortality rates and the development and progression of cancer, diabetes, hypertension, cardiovascular disease, depression, and obesity [18,19,20]. The mechanisms involved in the development of these diseases remain poorly understood [20,21]; however, exercise is involved in some metabolic pathways related to oxidative stress, immune responses, ECS, the conformation of intestinal microbiota, and the KP [22], which elicit adaptive changes which are observed, particularly, in myocytes. In turn, increasing mitochondrial biogenesis rates, anti-oxidant capacity, and protein synthesis, which generate a “pre-conditioned” state that protects cells from stressors that may occur during exercise, such as oxidative stress, increased temperature, and muscle atrophy, among others [23]. Particularly, some effects derived from physical exercise have been reported in the brain and the periphery, which positively influence the progression, clinical presentation, and prevention of various neurological, psychiatric, and metabolic diseases, including depression [24] and obesity [25].

### 2.1. The Kynurenine Pathway

**Figure 1:**
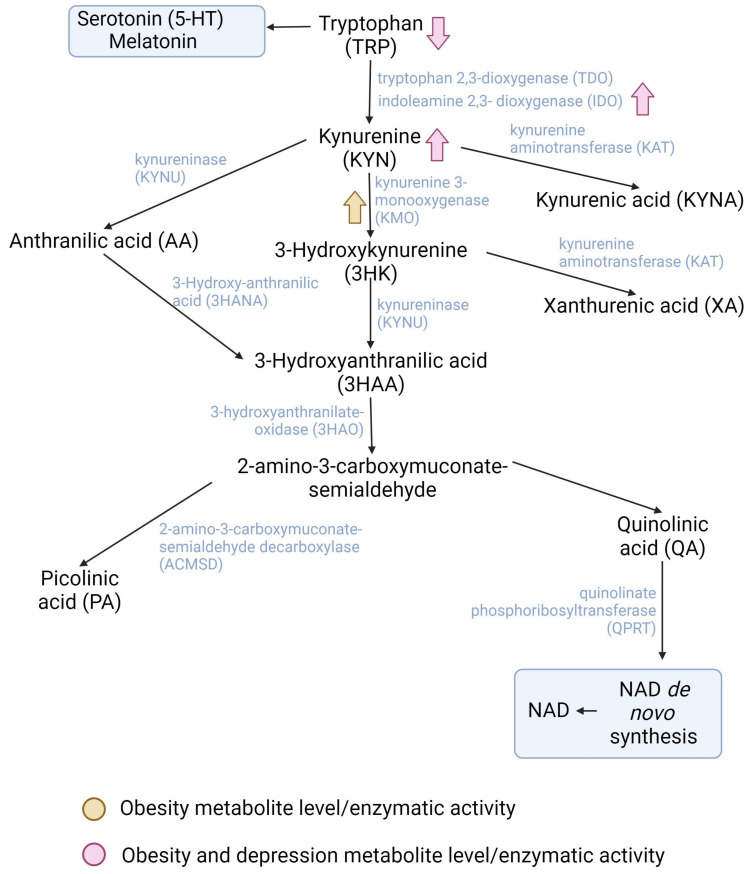
Main dysregulated enzymes and metabolites of the kynurenine pathway in obesity (orange) and both obesity and depression (pink). Created with BioRender.com.

Tryptophan (TRP) is an essential amino acid which is critical for protein synthesis; it is a precursor of the neurotransmitter serotonin, the hormone melatonin, and kynurenine catabolites, which are involved in the synthesis of the cellular cofactor nicotinamide adenine dinucleotide (NAD+). TRP is bound to albumin, either in plasma or serum, and only 5–10% is free to be immediately taken up by tissues and organs; thus, the availability of the KP relies on free TRP [11]. TRP is metabolized mainly through two metabolic pathways: the KP and the serotonin (5-hydroxytryptamine, 5-HT) pathway. A proportion of 95% of TRP comes from the diet; it is degraded through the KP to produce NAD, which is the preferred end product of the KP, and an essential cofactor which is necessary in maintaining energy metabolism [11] (Figure 1).

Skeletal muscle is the main reservoir of amino acids, which, under critical conditions, can be displaced for energy production by some organs [26] and contributes to KYN metabolism, and its composition and performance can be modified through exercise. In muscle fibers, there is a low expression of indoleamine 2,3-dioxygenase (IDO) and minimal tryptophan 2,3-dioxygenase (TDO) enzymatic activity, so the KYN produced in the liver or immune cells and circulating TRP are imported into muscle fibers through the large neutral amino acids transporters (LATs) following an open competition with other amino acids [27]. The most abundant enzymes within muscle fibers are KATs, KYNU, KMO, and 3-hydroxyanthranilate 3,4-dioxygenase (3HAO). KMO and 3HAO require molecular oxygen for their catalytic activity, whereas KATs and KYNU require α-keto acids. Therefore, during exercise, the increased O_2_ consumption required to sustain higher energy production from mitochondria could affect the KP by reducing the enzymatic activity of KMO and 3HAO and by inhibiting a class of prolyl-hydroxylases (PHDs); these utilize α-ketoglutarate as a substrate under hypoxia conditions, leading to the use of α-ketoglutarate availability by KATs [28]. Chronic endurance exercise increases skeletal muscle expression of all four KATs in humans through the PGC-1α1-PPARα/δ pathway to convert KYN to KYNA, elevating the serum concentration [29]. However, it remains unknown how KYNA leaves muscle fibers [30]. KYNA does not cross the brain–blood barrier (BBB), so decreasing KYN levels in the brain could reduce the stress-mediated effects underlying depressive symptoms [31]. PGC-1a co-activators and its variants PGC-1a1 and PGC-1a4 modulate processes related to energy metabolism and muscle mass maintenance by regulating physiological adaptations to exercise and promoting myokine secretion to regulate systemic energy expenditure [32]. Therefore, while PGC-1a1 regulates bioenergetic cellular processes, PGC-1a4 modulates muscle hypertrophy by repressing myostatin expression [27,33].

#### 2.1.1. Participation of Exercise-Induced Kynurenines in Obesity

Obesity is defined as abnormal and excessive fat accumulation with negative impact on health and quality of life [1]. It is a disease associated with the stimulation of uncontrolled inflammatory responses in white adipose tissue (WAT), muscle, liver, and pancreatic islets [34], resulting in chronic low-grade systemic inflammation (cLGI) that contributes to IDO and KP activation [35,36,37]; this is accompanied by a lack of physical exercise and excessive food intake with the consequent excess of TRP. In obesity, there is an aberrant metabolism of amino acids, specifically of TRP [38]—with TRP comprising mature adipocytes in the WAT, which are responsible for the excessive circulation of KYN production. This is a result of the over-expression of IDO1 and vitamin B6 deficiency [39], which exacerbate obesity and insulin resistance. The elevated circulating levels of KYN impair lipid homeostasis via aryl hydrocarbon receptor/signal transducers and the activation of transcription 3/interleukin-6 signaling (AhR/STAT3/IL-6); this stimulates lipid deposition and affects insulin sensitivity in adipocytes [39]. Apparently, the latter induces a compensatory effect of the low-grade chronic inflammation present in obesity [40,41], since KYN acts as an immunosuppressive factor [42,43]. However, scientific evidence points out that KYN could be acting as an agonist promoting obesity and insulin resistance instead of ameliorating the inflammatory environment in the adipose tissue of individuals with obesity [39]. The levels of KYN, KYNA, and QA are associated with higher body mass index (BMI) [44]. KMO is not expressed in primary adipocytes but its presence in this tissue is probably a consequence of its expression by resident macrophages. In this sense, KMO activation via proinflammatory M1 macrophages could redirect the KP to produce 3-HK and XA instead of NAD+ [44]. The well-established biomarkers of KP and INF-γ activation are the KYN/TRP ratio and the neopterin released by activated macrophages [45].

When exercising, the increment of KATs in skeletal muscle increases circulating KYNA, which regulates energy expenditure, promotes an anti-inflammatory environment in adipose tissue [29], and induces the production of xanthurenic acid (XA); this forms a complex with insulin, producing a diabetogenic effect [46]. In a mouse model of diet-induced obesity, the chronic exogenous administration of KYNA halts body weight gain and reduces adiposity in the subcutaneous WAT through the activation at GPR35, which increases the expression of gene networks involved in thermogenesis through the process of browning, lipid metabolism, and type 2 anti-inflammatory immune responses. In exercised mice, a daily elevation of KYNA occurs over a period of 1–4 weeks of exercise, resulting in weight loss due to the reduction in adipose generation [29] and the increase in adipose tissue energy expenditure through the browning of WAT occurring in the subcutaneous and visceral adipose deposits; here, the brown adipocytes waste energy by linking with fatty acid oxidation, which dissipates energy in the form of heat [47].

#### 2.1.2. Participation of Exercise-Induced Kynurenines in Depression

Depression is a chronic and debilitating medical illness that affects mood, thoughts, and physical health, with an important impact on quality of life for many people worldwide [48]. The exact neuronal mechanism of depression remains unknown, but there is an imbalance of various neurotransmission systems, such as monoamines and amino acids, specifically in glutamate transmission and/or decreased synaptic plasticity [49,50,51]. This is a result of a stress-induced neuroinflammatory pathway, which represents the link between depression and chronic stress [52]. In particular, the KP is activated by stress and inflammatory factors [53,54]. Recently, the participation of KYN in depression has gained great interest because, beyond just representing another simple marker of inflammation, the metabolites of KP—due to their neuroactive properties (on glutamate and cholinergic receptors)—are capable of modulating different neurotransmission systems; these can explain the different manifestations and clinical symptoms that are present in depression [55]. This participation has been recently supported by therapeutic interventions focused on inflammation, NMDA antagonists (ketamine), and probiotics; in addition to their positive adjuvant effects on depressive symptoms, these positive effects are related to changes in serum KYN [56,57,58]. However, it is also prudent to point out that some established therapeutic measures for the management of depression or refractory depression, such as electroconvulsive therapy or repetitive transcranial magnetic stimulation, have not been shown to significantly affect serum KYN levels; more studies are needed in this regard [59,60]. Furthermore, there is evidence that supports the role of KYN in bipolar disorder, where changes in serum KYN patterns have been documented in relation to the stage of the disease (depression vs. mania) [61], as well as in depression associated with other medical conditions such as post-stroke depression or depression in type 2 diabetes [62,63].

Physical exercise is an effective therapy that improves depressive symptoms [64]. The effects of physical exercise on peripheral KP metabolites occur when exercise is performed acutely and results in peripheral clearance of KYN, preventing its passage through the BBB [31]. In a study with healthy male adult participants (mean age 28.7), who performed ten intervals of one-minute high-intensity running followed by two minutes of low-intensity running for four consecutive days or over four weeks, concentrations of TRP and KYN decreased in plasma after exercise, and the downstream metabolites KYNA, 3-HK, and PA increased in the cerebrospinal fluid [65]. The increase in KYNA seems to be related to the over-expression of the PGC1-α and PPARα genes, which causes an increase in the expression of muscle KATs, as demonstrated in individuals who performed resistance exercises vs. controls [21]. On the other hand, PA cerebrospinal levels were found to be lower among patients with depression compared with subjects without depression [66]. It is noteworthy that, although these mechanisms have been demonstrated in animal models and in healthy humans, it could not be proven that physical exercise could induce long-lasting changes in the peripheral blood levels of KYNA; this was the case in a 12-week exercise intervention test performed among depressed patients [67], as well as in testing samples of the sweat of adult patients with chronic back pain following 28 days of training [68]. However, following 6 months of different swimming intensities, a peripheral overactivation of the KP has been observed, leading to a decrease in KYN levels and an increase in circulating KYNA and 3-HK levels [69].

### 2.2. The Endocannabinoid System

**Figure 2:**
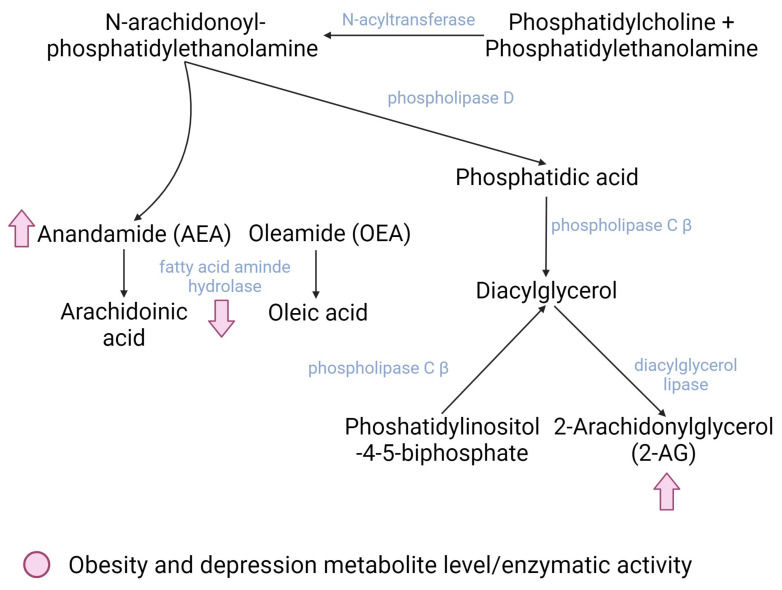
Main dysregulated enzymes and metabolites of the endocannabinoid system in both obesity and depression (pink). Created with BioRender.com.

The ECS is an endogenous signaling system that plays a central role in the development and function of the nervous system [70]. The ECS is formed by multifunctional components such as cannabinoid receptors (mainly CB1 and CB2, as these are the most frequently studied), their endogenous ligands, endocannabinoids (eCB), and several proteins involved in the transport, synthesis, and degradation of other neurotransmitters [70]. eCBs are derived from neural membrane lipids (i.e., phosphatidylcholine, phosphatidylethanolamine, and phosphatidylinositol-4,5-bisphosphate) that are released and can diffuse to neurons, microglia, and astrocytes. The most studied eCBs to date are 2-arachidonoylglycerol (2-AG), N-arachidonoyl-ethanolamine, anandamide (AEA), and oleamide (OEA), but new molecules related to cannabinoids have been emerging in the literature [68]. eCBs bind to their receptors, mainly CB1 receptors in neurons and muscle cells and CB2 receptors in microglia and peripheral immune cells, where they exert their modulatory functions on diverse cellular processes [71] (Figure 2).

There are two main pathways for the biosynthesis of eCB. The first one involves the transfer of an arachidonic acid from phosphatidylcholine to phosphatidyl ethanolamine; this is mediated by the enzyme N-acyltransferase, which generates N-arachidonoyl-phosphatidyl ethanolamine (NAPE), an endocannabinoid precursor. NAPE is further degraded to AEA and phosphatidic acid. Phosphatidic acid is then degraded to diacylglycerol (DAG) by the phospholipase C β enzyme (PLCβ) that can also degrade phosphatidylinositol-4,5-bisphosphate (PIP2) to DAG, which constitutes the second route of endocannabinoid synthesis. Finally, DAG is hydrolyzed to 2-AG; this is mediated by the diacylglycerol lipase enzyme [70]. The degradation of AEA occurs primarily through the fatty acid amino hydrolase enzyme (FAAH), whereas 2-AG is degraded mainly through three enzymes: monoacylglycerol lipase (MGL) and alpha/beta domain hydrolases 6 and 12 (ABHD6 and 12) [70].

Among people with obesity, the key role of the ECS is the overproduction of eCBs by adipose tissue; this raises their central and peripheral circulating levels and is probably due to the higher availability of eCB precursors and eCB catabolism dysfunction. The attachment of eCb to CB1 increases hunger and willingness to intake food, decrease peristalsis, and delays stomach emptying [72]; however, the CB1 blockade precludes the attachment of AEA—the natural antidepressant endocannabinoid—resulting in depression symptoms [73].

Therefore, there is growing interest in determining the role of the ECS in health and disease processes because this system exerts several effects; in turn, it is influenced by many other signaling pathways involved in various physiological processes. The interest in generating more knowledge in the exercise-derived endocannabinoidome has been driven mainly because several of its participants have been considered as emerging targets for innovative therapeutic approaches [74].

#### Involvement of Exercise-Derived Endocannabinoids in Obesity and Depression

The participation of eCB and related enzymes in obesity has been recently highlighted. Over-stimulation of CB1 increases food intake and inflammation whereas activation of CB2 decreases the food intake, limits body weight gain, and inhibits inflammatory processes [75,76]. Results of some preclinical studies obtained from rodents indicate that the ECS, as an important component of the gut–brain axis, is dysregulated among people with obesity and can control food intake through eCB-mediated gut–brain signaling, which could represent a therapeutic strategy for the treatment of obesity and related metabolic disorders in humans.

eCB can be synthesized as a consequence of high-intensity physical exercise or an acute boost of physical exercise (age-adjusted maximum heart rate) in recreationally fit human runners at four different intensities, incrementing their circulating concentrations [68,77], which, in turn, decreases the perception of pain (anti-nociceptive responses), displays protective effects on mood disorders, induces mild sedation, analgesia, increases euphoric feelings (endorphins), and exerts anti-anxiolytic effects, as demonstrated in sport practitioners for a variable period of time after exercise [78]. The increment of circulating eCB has been related to CB1 activation in the liver and adipose tissue, and the generation of eCB in skeletal muscle to regulate metabolism and restore energy consumption that follows exercise [10]. Moreover, AEA and other N-acylethanolamines (NAEs) have been suggested to elicit mitochondrial biogenesis via activation of PPARγ, enhanced glucose uptake, and improved insulin action in the skeletal muscle; AEA could act as vasodilator evoking a hypotension state that may facilitate blood flow during exercise [79]. The positive correlation between the increase in circulating AEA and BDNF levels after a 90 min exercise in healthy cyclists could explain some effects of exercise on cognitive functions and mood, suggesting that eCB could mediate the exercise-induced increases in BDNF levels and CB1 regulation [80]. Therefore, many attempts have been made to develop and introduce exercise-based programs to promote neuroplasticity and improve motor dysfunctions, some physiologic alterations observed in obesity, and some psychiatric symptoms reported in depressive patients [78].

During the past decade, due to the increased demand for effective anti-obesity drugs rather than exercise routines, the use of the drug rimonabant—which is a selective antagonist of CB1 involved in the regulation of food intake—was shown to lead to significant weight loss compared to the placebo group. However, those patients who received rimonabant showed severe adverse trends toward symptoms of depressed mood and anxiety; this led to alertness among those prescribing weight loss agents, as it highlighted the role of the ECS in mood and emotion modulation. As of the time of writing, rimonabant has been discontinued for human consumption [81]. There are some phytocannabinoids such as cannabidiol (CBD) and D9-Tetrahydrocannabivarin (THCV), that are involved in improving hyperglycemia and inhibiting appetite, respectively. These actions make them very interesting molecules which, alone or in combination, have potential to bring about benefits in the treatment of obesity and diabetes; this is because their pharmacological profiles are distinct from that of rimonabant, and therefore they will have different side effects [82]. 

Conversely, treatment with regulated prescription and preferred exercise, supervised by physical therapists specialized in sport medicine, has been shown to lead to beneficial mood outcomes; increases in AEA and 2-AG concentrations have been reported among highly active individuals, with slow jogging (30 min treadmill run) and medium-intensity workouts. Interestingly, higher circulating levels of AEA, but not 2-AG, were found in the plasma of human participants post run compared to the levels among pre-exercise samples from volunteers; the difference between pre- and post-exercise AEA levels positively correlated with the differences between positive pre- and post-exercise effects (enthusiasm, energy, and pleasurable engagement) [83].

## 3. Crosstalk between the KP and the ECS in Obesity and Depression during Exercise: Potential Functional Interactions

**Figure 3:**
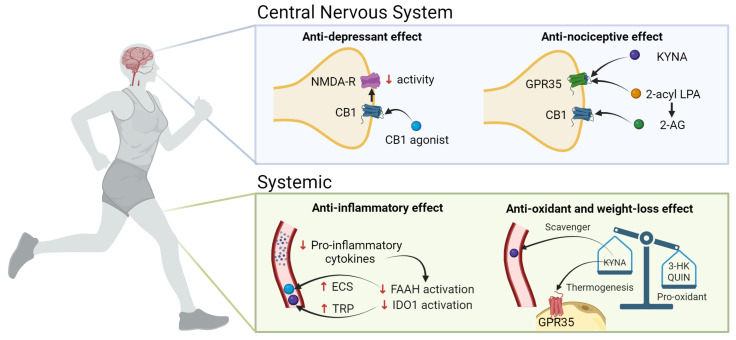
Exercise-induced modulation of the KP and the ECS capable of ameliorating both depressive symptoms and weight loss. 2-AG, 2-arachidonoyl glycerol; 3-HK, 3-hydroxykynurenine; CB1, cannabinoid receptor 1; KP, kynurenine pathway; ECS, endocannabinoid system; FAAH, fatty acid amino hydrolase; GPR35, G protein-coupled receptor 35; IDO,1 indoleamine 2,3-dioxygenase; KYNA, kynurenic acid; LPA, lysophosphatidic acid; NMDA-R, N-methyl-D-aspartate receptor; QUIN, quinolinic acid; TRP, Tryptophan. Created with BioRender.com.

The ECS and the KP have been strongly implicated in obesity and depression, and the link between these two systems is via chronic low-grade systemic inflammation in obesity and the neuroinflammatory and dysregulated serotonergic component in depression [22]. Combined, these results constitute a novel challenge in the search of more and yet-unknown biologically relevant interactions between these systems; however, this evidence must be demonstrated in future clinical trials (Figure 3).

### 3.1. NMDAr and CB1

The co-localization of receptors is critical because it allows for protein coupling to occur between signaling cascades from different pathways. NMDA and CB1 receptors undergo pre- and post-synaptic co-localization [84], and the effects of this co-localization result in the opposite glutamatergic NMDA function through the reduction in glutamate release upon cannabinoid binding to CB1 (pre-synaptic) and the NMDA-elicited calcium increase which is secondary to endocannabinoid receptor antagonists (post-synaptic) [85]. Glutamate is the main ligand for NMDAr and the activation of this receptor is involved in synaptic processes. On the other hand, when CB1 is activated, mainly by 2-AG or AEA, the intracellular levels of Ca2+ increase, whereas the levels of K+ decrease in presynaptic neurons, inhibiting the release of glutamate to the synaptic space and affecting the functions of postsynaptic neurons [34,85]. The involvement of cannabinoids in decreasing the activity of NMDAr when binding to CB1 has been elegantly reviewed by some authors [12,85], but also it has been reported that ketamine, an antagonist of NMDAr, is effective as an antidepressant drug. However, this potential therapeutic effect has not been demonstrated for other NMDAr antagonists [86].

### 3.2. GPR35 and KYNA

In addition to CB1 and CB2, eCBs can act as the agonists to many G-protein-coupled receptors (GPRs) [87]. Particularly, GPR35 shares several structural and functional properties with cannabinoid receptors; it co-localizes and co-expresses with CB1 receptors [88] and can act as an endogenous receptor for KYNA, inducing anti-nociceptive effects in the nervous system [4,89]. This nociceptive function may represent a novel interaction between ECS and the nervous system, as modulated by KYNA levels [88]. Furthermore, the mediator 2-acyl lysophosphatidic acid can be enzymatically converted to 2-AG, which binds to CB1 and CB2; 2-AG is able also to bind to GPR35. KYNA activates GPR35 in mice, regulating adipose tissue energy homeostasis which stimulates the expression of lipid metabolism and thermogenic and anti-inflammatory genes in the adipose tissue. In human adipose tissue, GPR35 expression correlates with genes involved in transcriptional regulation of adipocyte browning, an event that can be explored as a therapeutic target for obesity [29]. The long-term systemic administration of KYNA to the brain of rats specifically elevates the abundance of functional CB1 receptors in them, which can be a compensatory mechanism on the ECS induced by the long-term KYNA exposure [90]. In this sense, it seems that GPR35, cannabinoid receptors, ECS, and the KP are linked by intrinsic ligand conversions [89].

### 3.3. Inflammation

Inflammation could explain some of the changes in the KP among people with obesity and depression [91]. The adipose tissue is the primary source of proinflammatory cytokines such as IL-6, tumor necrosis factor (TNF), and interferon gamma (IFN-γ) during inflammatory processes [92,93]. The adipose tissue is crucial in the study of obesity and is considered a risk factor for the development of other pathologies. Among patients with obesity, the KP is activated and the expression of the IDO1 gene is increased, eliciting the conversion of TRP to KYN [6] and the association of CRP (C-reactive protein) with high levels of QA and low KA/QA ratio; this reflects the proinflammatory and neurotoxic effects of QA and anti-inflammatory effects of KYNA [94]. Nevertheless, among people with depression, neither CRP nor BMI explain the differences when compared to healthy people [91]. The hypothesis that the interactions between ECS and KP metabolites could have clinical implications started with the finding of positive associations among some of these metabolites, particularly 2-AG, with IL-6 registered in patients with some psychiatric disorders compared to volunteers without these psychiatric disorders. The authors demonstrated relevant associations between IL-6 levels and the individual risk preference test and between PA levels and the test for neuroticism [95]. It has been suggested that inflammatory cytokines could be responsible for influencing eCB deposits among women with obesity, which, in turn, activate the ECS; in contrast, the expression of FAAH, which degrades the AEA in abdominal adipose tissue, is decreased, inhibiting the degradation of eCB [7]. FAAH and IDO1 enzymatic activities have been proposed to be able to promote depressive symptoms; therefore, these enzymes represent a potential therapeutic target in this disease [96]. Hence, the modulation of the proinflammatory condition, particularly in adipose tissue, can regulate both the ECS and KP and might improve depression-associated symptoms.

Also, these proinflammatory cytokines can cross the BBB [97] through specific cytokine transporters and circumventricular organs and reach the brain [98]. Additionally, under the physiological context of low-grade inflammation—as seen in obesity—there can be a loss of the BBB’s integrity that can increase the infiltration of these cytokines, promoting an inflammatory condition in the CNS that impacts the progression of neurologic and psychiatric diseases [99]. Thus, the modulation of the KP via CB2 activation has been suggested as a potential target to counteract chronic inflammation and its associated effects concerning some diseases in the CNS. The CB2 stimulation has been linked to decreased levels of various proinflammatory cytokines, such as IFN-γ, which typically increase IDO activity [22]. In addition, upon its activation, CB2 increases anti-inflammatory cytokine levels, including those of interleukin-10 (IL-10) [100], and these increments in IL-10 can effectively interfere with IDO activity [101].

### 3.4. Oxidative Stress

Some of the KP metabolites can exert cytotoxic functions that favor either the generation of oxidative stress or protect cells from these oxidant molecules. 3-HK inhibits complexes I, II, and IV of the electron transport chain [102] and promotes the generation of reactive oxygen species [103]. However, 3-HK has been described also as a free radical scavenger [104]. QUIN generates oxidative stress by excitotoxicity [105], while KYNA exhibits scavenger activity [106,107]. On the other hand, there is evidence that CB1 activation promotes oxidative stress [108]; however, its neurological effects are mainly positive, showing antidepressive, anti-inflammatory, enhanced memory, and neuroplasticity improvements [109].

Finally, there are two research areas that we would like to put forward for further investigation which will provide a better understanding of the topic of this review: the use of phytocannabinoids combined with physical exercise as an alternative treatment of obesity and depression; research to determine why physical exercise could not induce long-lasting changes in the KP metabolites in both health and illness.

## 4. Conclusions

The endocannabinoid system and the kynurenine pathway can interact with each other through their metabolites—endocannabinoids and kynurenines—which are released during acute exercise and which are capable of mediating exercise-derived benefits among people with obesity and depression [110,111]. Many of the benefits of physical exercise, especially in the context of diseases, can be attributed to the action of eCBs and kynurenines in their signaling tissues and the activation of downstream pathways, or by their metabolic transformation to other molecules [112]. Clear evidence of these benefits remains limited, because many possible outcomes (aerobic capacity, adverse effects, and quality of life) have not been included in the corresponding research [113]; however, the interaction of both systems can improve understanding of why exercise is helpful in these diseases. Finally, considering the failure of rimonabant as a treatment for obesity (the treatment led to significant weight loss but had severe adverse trends toward depressed mood and anxiety), we suggest that exercise-derived endocannabinoidomes and kynurenines offer potential therapeutic targets. In this recommendation, we place emphasis on the increment of KYNA, AEA, and 2-AG circulating levels, which have significant benefits in both obesity and depression: (a) KYNA induces an anti-inflammatory environment in the adipose tissue, preventing weight gain and rescuing adiposity in subcutaneous WAT through GPR35 receptors and KYN clearance in the periphery; this reduces depressive symptoms. (b) AEA and 2-AG, together with other myokines, such as BDNF, improve cognitive functions and mood.

## Data Availability

The data presented in this study are available on request from the corresponding author.
